# Orthodontic movement in deciduous teeth

**DOI:** 10.1590/2176-9451.20.2.016-019.oin

**Published:** 2015

**Authors:** Alberto Consolaro

**Affiliations:** 1 Full professor, School of Dentistry - University of São Paulo (USP)/Bauru and School of Dentistry - University of São Paulo (USP)/Ribeirão Preto.

**Keywords:** Root resorption in deciduous teeth, Orthodontic movement, Exfoliation, Eruption, Anchorage, Orthopedics

## Abstract

Deciduous teeth exfoliate as a result of apoptosis induced by cementoblasts, a process that reveals the mineralized portion of the root while attracting clasts. Root resorption in deciduous teeth is slow due to lack of mediators necessary to speed it up; however, it accelerates and spreads in one single direction whenever a permanent tooth pericoronal follicle, rich in epithelial growth factor (EGF), or other bone resorption mediators come near. The latter are responsible for bone resorption during eruption, and deciduous teeth root resorption and exfoliation. Should deciduous teeth be subjected to orthodontic movement or anchorage, mediators local levels will increase. Thus, one should be fully aware that root resorption in deciduous teeth will speed up and exfoliation will early occur. Treatment planning involving deciduous teeth orthodontic movement and/or anchorage should consider: Are clinical benefits relevant enough as to be worth the risk of undergoing early inconvenient root resorption?

## Abstract (pt)

O dente decíduo é esfoliado graças à apoptose em seus cementoblastos, que desnuda a parte mineralizada da raiz e atrai os clastos. A rizólise é lenta, pois faltam mediadores em quantidade para acelerar o processo, mas ela se acelera e unidireciona quando se aproxima um folículo pericoronário de dente permanente rico em EGF e outros mediadores da reabsorção óssea - os responsáveis pelas reabsorções óssea na erupção e dentária decídua na rizólise e esfoliação. Se houver movimentação ortodôntica ou ancoragem em dentes decíduos, aumenta-se, também, o nível local desses mesmos mediadores, devendo-se estar bem consciente de que *haverá uma aceleração da rizólise e, em decorrência, uma antecipação de sua esfoliação*. No planejamento de casos em que dentes decíduos estejam envolvidos na movimentação ortodôntica e/ou ancoragem, deve-se ponderar: o benefício clínico para o paciente será relevante, a ponto de valer o risco de uma rizólise abreviada e inconveniente?

Deciduous teeth are preferably termed "temporary" or "primary" by Anglo-Saxon authors, as they consider "deciduous" a popular and rather lay term. Nevertheless, it perfectly describes the conditions of such teeth, as it comes from Latin "that which falls!"

## INDUCTION AND ACCELERATION OF ROOT RESORPTION IN DECIDUOUS TEETH AND TOOTH ERUPTION

Once deciduous teeth are completely formed, their cells immunohistochemically reveal that they are gradually dying by apoptosis. The latter is the process of programmed cell death that occurs as a result of release of p53 gene which, in turn, initiates a series of intracellular biochemical phenomena that lead to cytoskeleton breakage, as well as cell nucleus and membrane degradation. 

Within a few hours or days, the cell releasing the p53 gene shrinks and starts losing fragments as if it were a flower dropping its petals or a tree losing its leaves. As odontoblasts and cementoblasts die here and there, they leave the surface of deciduous teeth with little, numerous, growing cell-free areas. Odontoblasts and cementoblasts protect odontogenic mineralized tissue surface against clasts - typical cells of mineralized tissue resorption, regardless of its nature.

**Figure 1 -:**
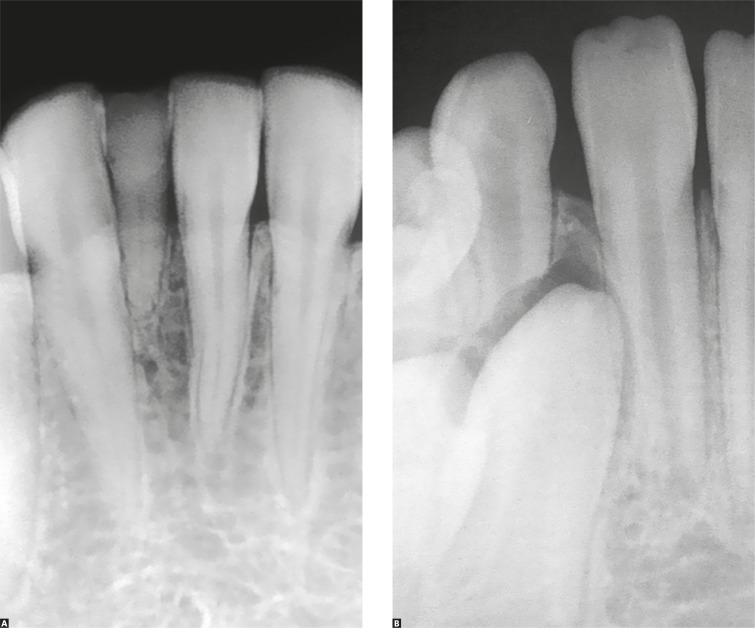
Despite the absence of a permanent tooth to come, in A, root resorption slowly occurs in deciduous teeth. Due to being too near the pericoronal follicle, in B, numerous mediators accumulate and, as a result, speed up and cause mineralized tissue resorption to move in one single direction, including deciduous teeth roots.

As a result of apoptosis, cementum and dentin receive and fix clasts in surface and cell-free areas, thereby triggering root resorption in deciduous teeth. This process occurs regardless of root region, whether apical, medium or cervical (Fig 1). Root resorption in deciduous teeth is initially triggered, induced and caused by apoptosis of deciduous teeth cementoblasts and odontoblasts.

Apoptosis is one of the most exquisite and impressive mechanisms the body develops to get rid of unwanted cells, such as those achieving their highest functional peak and which have already accomplished a specific mission, or rebel and defective cells willing to acquire autonomy within the body and, should they survive, produce cancerous clones. All cell tissues are subjected to apoptosis; without it, we would have a much briefer life.

Although root resorption in deciduous teeth takes place simultaneously in all root surfaces, including the inside of the root, it is likely to speed up and spread in one single direction in certain regions too near the permanent tooth to come (Fig 1). Permanent tooth pericoronal follicle is rich in growth factors typical of reduced enamel epithelium and dental lamina remnants, both of which are present in connective tissue.

The reduced epithelium has already produced the enamel and is now tightly attached to it which, in turn, is nourished by capsular connective tissue, forming the pericoronal follicle. The major structure responsible for tooth eruption is the pericoronal follicle rich in epithelial growth factor (EGF). EGF induces epithelial cell proliferation in order to preserve the epithelial tissue - a structure under continuous renewal. Meanwhile, numerous EGF molecules act in the surrounding bone tissue, inducing pericoronal bone resorption and leading the way to the development of the new tooth into the oral cavity.

As the pericoronal follicle and its mediators controlled by the EGF promote pericoronal bone resorption in the eruption pathway, it speeds up the slow process of root resorption per se whenever it is near a deciduous tooth. For this reason, the process of resorption is established in deciduous roots and turned towards the region of the permanent tooth to come. 

Whenever permanent and deciduous teeth are near each other, the gap between them is filled with follicular tissue adhered to the enamel by means of the reduced epithelium on one side, and connective tissue rich in clasts near the surface of the deciduous tooth on the other side. The presence of a permanent tooth to come does not induce root resorption in deciduous teeth, but rather speeds the process up and encourages it to spread in one single direction (Fig 1)!

In short:

1) Deciduous teeth exfoliate as a result of cell death by apoptosis which, in turn, is induced by cementoblasts and odontoblasts. This process reveals the mineralized portion of the root while attracting clasts. Root resorption in deciduous teeth takes place throughout the entire root surface. It is a slow process due to lack of mediators necessary to speed it up.
2) Root resorption in deciduous teeth accelerates and spreads in one single direction whenever a pericoronal permanent tooth follicle, rich in epithelial growth factor (EGF) or other bone resorption mediators, come near.
3) Pericoronal follicle mediators are responsible not only for root resorption during eruption, but also for deciduous teeth root resorption and exfoliation.

## ORTHODONTIC MOVEMENT RELIES ON MEDIATORS

Whenever 0.25-mm thick periodontal ligament of which 50% volume is composed by vessels, is compressed, stress and/or inflammation are induced. Both processes are characterized by local accumulation of bone resorption mediators on periodontal alveolar bone surface.

Mediators, such as cytokines, growth mediators and prostaglandins, excite local bone resorption as they meet, attach or interact with membrane receptors in osteoblasts, macrophages and clasts. Together, these cells are known as BMU or bone modeling units. 

Cementoblasts covering the root surface of permanent teeth lack receptors of local and systemic mediators of bone resorption. Thus, the tooth moves amidst bone structures, inducing resorption while remodeling periodontal tissues without causing root resorption. 

In short,^1^ permanent teeth have their roots preserved by cementoblasts destitute of receptors of resorption mediators: *cementoblasts protect the root against tooth resorption*. Mediators are present, but do not interact with cementoblasts, only with osteoblasts and associated cells. 

That is the reason why teeth do not undergo root resorption when forces do not fully compress the vessels at the site where they act on periodontal ligament. Meanwhile, whenever movement is induced by extremely concentrated intense forces, cementoblasts might die by anoxia. Additionally, root surfaces will be subjected to resorption, even if temporarily.

## ORTHODONTIC MOVEMENT IN DECIDUOUS TEETH!

In an orthodontic and/or orthopedic context, applying forces of any nature over deciduous periodontal ligament promotes stress and inflammation, as observed in permanent periodontal ligament. Likewise, there will be accumulation of mediators and bone resorption will take place on the periodontal surface of alveolar bone. 

Nevertheless, as bone resorption mediators accumulate on periodontal ligament compressed under stress and/or inflammation; osteoblasts, clasts and macrophages organized in BMU are encouraged to attach to exposed root surfaces of the deciduous tooth. 

At this point, the root surface of completely formed deciduous teeth are destitute of cementoblasts, as the latter died by apoptosis. Mineralized structures directly exposed to the connective tissue attract or promote chemotaxis of clasts, especially when excited by mediators of bone resorption accumulated as a result of compression of vessels and hypoxia. This process is typical of orthodontic movement. 

Root resorption of deciduous teeth is expected to speed up when orthodontic movement takes place. Importantly, the former is inherent to the latter. Whenever a physiological structure, such as the permanent tooth pericoronal follicle permeated by mediators of bone resorption, is too near deciduous roots lacking cementoblasts, root resorption will be inevitably sped up (Fig 1). Likewise, whenever orthodontic movement takes place, deciduous teeth periodontal ligament will present with great local concentration of mediators of mineralized tissue resorption on both surfaces: bone and root.

## FINAL CONSIDERATIONS

Should there be an opportunity or need to subject deciduous teeth to orthodontic movement or anchorage for orthopedic purposes, one should be fully aware that root resorption will speed up and exfoliation will early occur. 

Treatment planning involving deciduous teeth orthodontic movement and/or anchorage should consider: *Are clinical benefits relevant enough as to be worth the risk of undergoing early inconvenient root resorption?*
